# Strategies to increment *in vivo* and *in vitro* embryo production and transfer in cattle

**DOI:** 10.21451/1984-3143-AR2019-0042

**Published:** 2019-10-23

**Authors:** Gabriel A. Bó, Andrés Cedeño, Reuben J. Mapletoft

**Affiliations:** 1 Instituto de Reproducción Animal Córdoba (IRAC), Zona Rural General Paz, (5145) Córdoba, Argentina.; 2 Instituto de Ciencias Básicas, Medicina Veterinaria, Universidad Nacional de Villa María, Villa del Rosario, Córdoba, Argentina.; 3 Doctorado en Ciencias Mención Agroalimentos, Universidad Nacional de Villa Maria, Villa del Rosario, Córdoba, Argentina.; 4 Laboratorio de Biotecnologías de la Reproducción Animal, Medicina Veterinaria, Escuela Superior Politécnica Agropecuaria de Manabí (ESPAM), Calceta, Manabí, Ecuador.; 5 Western College of Veterinary Medicine, University of Saskatchewan, Saskatoon, SK, Canada.

**Keywords:** superstimulation, bovine embryos, fixed-time embryo transfer

## Abstract

Knowledge of follicular wave dynamics obtained through the use of real-time ultrasonography and the development of the means by which follicular wave dynamics can be controlled have provided practical approaches for the *in vivo* and *in vitro* production and transfer of embryos in cattle. The elective control of follicular wave emergence and ovulation has had a great impact on the application of on-farm embryo transfer, especially when large groups of donors need to be superstimulated at the same time. Although estradiol and progestins have been used for many years, practitioners in countries where estradiol cannot be used have turned to alternative treatments, such as mechanical follicle ablation or the administration of GnRH for the synchronization of follicle wave emergence. *In vitro* embryo production also benefits from the synchronization of follicle wave emergence prior to Cumulus Oocyte Complexes (COCs) recovery. As *Bos indicus* cattle have high antral follicle population, large numbers of oocytes can be obtained by ovum pick-up (OPU) without superstimulation. However, synchronization of follicular wave emergence and superstimulation is necessary to obtain high numbers of COCs by OPU and blastocysts following *in vitro* fertilization in *Bos taurus* donors. Finally, embryos can now be transferred in commercial beef or dairy herds using efficacious synchronization and re-synchronization protocols that are easily implemented by farm personnel. These technologies can also be used to resolve reproductive problems such as the reduced fertility observed during summer heat stress and/or in repeat-breeder cows in commercial dairy herds.

## Introduction

The objective of ovarian superstimulatory treatments in cattle is to stimulate the growth of the maximum number of antral follicles that produce competent oocytes (Bó and Mapletoft, 2014). The usual regimen for *in vivo* embryo production has been twice daily intramuscular (i.m) treatments with FSH for 4 or 5 days (Bó and Mapletoft, 2014). However, previous experiments have indicated that follicle maturation and ovulation rate can be improved in at least some donors if FSH treatments are administered over 6 or 7 days (Bó *et al*., 2008; García Guerra *et al*., 2012). For *in vitro* embryo production (IVP), the requirement of superstimulation with gonadotropins prior to ovum pick-up (OPU) is still under discussion, and the approach may differ depending on whether the donors are of *Bos taurus* or *Bos indicus* breedings. Regardless of the method of embryo production, these technologies can be implemented in commercial beef and dairy operations for genetic improvement and even to increase fertility during the summer heat stress and/or in repeat-breeder dairy cows. The objective of this manuscript is to briefly summarize the existing protocols for superstimulating donors for *in vivo* and *in vitro* embryo production and to propose alternatives by which embryo transfer technologies can be implemented more widely in commercial herds.

## Superstimulation and *in vivo* embryo production

Two very important factors influencing variability in superstimulatory response are the intrinsic number of antral follicles in donors, and the stage of follicular development at the beginning of FSH treatments. Response can be predicted by antral follicle count done with ultrasonography (Singh *et al*., 2004; Ireland *et al*., 2011), or measurement of circulating concentrations of anti-Müllerian hormone [AMH; *Bos taurus* (Rico *et al*., 2012; Monniaux *et al*., 2013), *Bos indicus* (Batista *et al*., 2014)]. High antral follicle counts have resulted in more ovulations and a greater number of transferable embryos following superstimulation with FSH than low antral follicle counts (Ireland *et al*., 2007). Similarly, the top quartile of circulating AMH values was associated with a greater superovulatory response than the lowest quartile (Souza *et al*., 2014). Therefore, selection of donors based on antral follicle counts or AMH concentrations may be important for predictable and economical embryo production.

## Synchronization of follicle wave emergence for superstimulation

Transvaginal ultrasound-guided follicle ablation followed by FSH treatments 1 or 2 days later isvery efficacious in the synchronization of follicle wave emergence (Bergfelt *et al*., 1997; Baracaldo *et al*., 2000; Lima *et al*., 2007), but requires specialized skills to apply in the field. However, if donors are housed in an embryo production facility, follicle aspiration by OPU can be used to obtain COCs for IVP and at the same time synchronize follicle wave emergence for the production of *in vivo-*derived (IVD) embryos in the same donor (Surjus *et al*., 2014). The number of embryos produced in that study was higher when the interval from OPU to superstimulation was 2 days rather than 1 day. However, there was a concern that the number of recruited 3 to 5 mm follicles 2 days after OPU was lower than those counted at the time of OPU itself, which was performed at random stages of the estrous cycle (15.2 ± 2.3 *vs* 33.7 ± 2.3; P < 0.05; Surjus *et al*., 2014). However, a more recent report (Cirit *et al*., 2019) suggested that this can be overcome using a longer superstimulation treatment (i.e., 6 days) rather than the traditional 4-day FSH protocol. In this study, donors received 400 IU of equine Chorionic Gonadotropin (eCG) 1 day after OPU followed by a 5-day FSH treatment initiated 1 day later (Cirit *et al*., 2019). However, a critical study with a representative number of animals and an adequate control group is needed to confirm this notion. The practical application of producing embryos *in vitro* and *in vivo* in succession in the same donor has important practical implications because it potentially increases the production of embryos in a short period of time.

The preferred approach for synchronization of follicular wave emergence in South America is the administration of 2 mg estradiol benzoate (EB) or 5 mg estradiol-17ß and 50 – 100 mg progesterone (P4) and insertion of P4-releasing device 4 days before initiating FSH treatments (Bó *et al*., 1996, 2002a; Bó and Mapletoft, 2014). This protocol has been extensively reviewed (Bó and Mapletoft, 2014) and will not be discussed further in this manuscript. However, estradiol is not available in many other countries around the world, requiring the use of alternatives such as follicle ablation or GnRH to synchronize follicle wave emergence prior to superstimulation (reviewed in Bó and Mapletoft, 2014)

Attempts to synchronize follicular wave emergence for superstimulation with GnRH were initially unsuccessful; however, subsequent field data were more promising. In these cases, GnRH was administered 1.5 to 3.0 days after the insertion of an intravaginal P4-device which may have increased the probability of an LH-responsive follicle at the time of treatment with GnRH. Indeed, Bó *et al.* (2010) reported the strategic use of PGF_2α_, a P4-device and GnRH to induce ovulation prior to initiating FSH treatments. Basically, a persistent follicle was induced by treatment with PGF_2α_ at the time of progestin device insertion; following administration of GnRH 7 days later, ovulation occurred in more than 95% of the animals. Superstimulation initiated 36 hours after GnRH (with the P4-device remaining in place) resulted in a superovulatory response that did not differ from controls superstimulated on Days 8 to 12 of the estrous cycle. More recently, Hinshaw *et al*. (2015) reported no difference in superovulatory response whether GnRH was administered 2 or 7 days after insertion of a P4-device.

## Extended superstimulatory treatment protocols

An earlier study provided rationale for the hypothesis that superstimulatory treatment may recruit follicles into the wave and allow small follicles to attain medium and large diameters (Adams *et al*., 1994). Based on this notion, attempts have been made to increase the superovulatory response by adding eCG treatment prior to initiating FSH treatments. Pre-treatment with eCG 2 days before the conventional FSH treatment protocol resulted in a numerically greater number of transferable embryos (6.7 ± 1.2 *vs* 4.9 ± 0.9) in an unselected group of donors (Caccia *et al*., 2000), and a significantly greater number of transferable embryos in donors that were defined as poor responders (3.6 ± 0.6 *vs* 1.0 ± 0.2; Bó *et al*., 2008).

A more recent study evaluated the superovulatory response and embryo recovery in donors treated with either a 4-day or a 7-day FSH treatment protocol utilizing the same total dose of 400 mg FSH (Folltropin-V; Vetoquinol Inc., Canada) administered twice daily at a constant daily dosage (García Guerra *et al*., 2012). The mean number of ovulations detected by ultrasonography was greater in the 7-day treatment group (30.9 ± 3.9 *vs* 18.3 ± 2.9, P = 0.01), consistent with a numerically greater number of follicles ≥ 10 mm just prior to ovulation (27.5 ± 4.1 *vs* 19.5 ± 2.6; P = 0.11). Moreover, ovulations occurred more synchronously in the 7-day group (93% of ovulations occurred 12 to 36 hours post-LH as compared to 66% in the 4-day group) suggesting that the superstimulated follicles were more mature and capable of responding to an LH stimulus. Although the total number of ova/embryos, fertilized ova and transferable embryos did not differ statistically, all end-points favored the 7-day group. In addition, when data from two cows with fertilization failure were removed, the number of transferable embryos tended to be higher in the 7-day group (7.6 ± 1.7 *vs* 4.2 ± 1.5; P = 0.07).

In another study (Dias *et al*., 2013a), a 7-day superstimulation protocol was used to investigate the influence of P4 on follicle growth, ovulation and oocyte competence. Beef cows were superstimulated with 25 mg of FSH twice-daily for 4 or 7 days. Again, the superstimulatory response (number of large follicles just prior to insemination) was greater (P < 0.05) in the 7-day group, and the numbers of ovulations (15.4 *vs* 11.6) and embryos (6.7 *vs* 5.9) were numerically higher in the 7-day group.

The duration of treatment rather than the FSH dose appears to be responsible for the increase in the superstimulatory response. In the two studies cited above, the number of ovulatory-sized follicles just prior to ovulation was greater following 7 days of superstimulation than 4 days, whether the total dose of FSH was greater (Dias *et al*., 2013a) or the same (García Guerra *et al*., 2012). In addition, there was noevidence that more follicles were recruited; the total numbers of follicles at the end of FSH treatment was the same as that at the beginning of FSH treatment, the only difference was the distribution of follicle sizes. Furthermore, in a recent study of follicles undergoing a 4-day superstimulation protocol, gene expression in granulosa cells was altered compared to a single, naturally occurring dominant follicle (Dias *et al*., 2013b, 2014). Expression of growth-related genes similar to the pre-LH stage of follicle growth (even though LH had been administered) and those involved in oxidative stress response were up-regulated in granulosa cells of follicles undergoing a 4-day FSH superstimulation protocol, compared to a preovulatory follicle of an unstimulated follicular wave. Genes related to a disturbance in angiogenesis were also up-regulated in superstimulated follicles. Since the mean growth rate of follicles between initiation of treatment and ovulation was more similar to naturally cycling cattle in the 7-day group than in the 4-day group, we speculate that gene expression during the 7-day superstimulation protocol may be more similar to the naturally occurring single preovulatory follicle.

## Use of eCG to replace the last four FSH applications

In search of possible improvements to the superstimulatory treatment protocol Price *et al.* (1999) demonstrated that during the superstimulatory treatment, LH pulse frequency diminish shortly after the first FSH injection and are accentuated during the last injections and the preovulatory period. This occurs as a consequence of the high steroidogenic activity and an increase in the concentrations of estradiol in superstimulated cows and may affect superovulatory response and embryo quality (Price *et al*., 1999). Therefore, a treatment that provides LH support at the end of the superstimulation treatment may be beneficial, since LH has been shown to be essential for the final growth of the superstimulated follicles and for the completion of oocyte maturation (Oliveira *et al*., 2014).

Equine Chorionic Gonadotropin is a complex glycoprotein that has FSH and LH activity in non-equid species (Murphy and Martinuk, 1991). A remarkable feature of eCG that has been exploited in multiple experimental and commercial contexts is its ability to express FSH and LH activity in the cow (Murphy, 2012). In cattle, this gonadotropin has a prolonged action time, due to the proportion of sialic acid (10 to 15%) present in its molecule (Murphy and Martinuk, 1991).

In the early days of bovine embryo transfer, eCG was used to induce superovulation in donors (Bó and Mapletoft, 2014). However, its long half-life, which was a feature for induction of superovulation with a single administration, resulted in multiple unovulated follicles and poor embryo quality at the time of embryo collection (reviewed in Bó and Mapletoft, 2014; Murphy, 2012). More recently, the last two doses of FSH in a superstimulation protocol have been replaced by different dosages of eCG, with the intention of providing more LH support to the growing follicles (reviewed in Barros *et al*., 2012). Some studies have shown beneficial effects of the association of FSH and eCG (Cifuentes *et al*., 2009; Reano *et al*., 2009; Mattos *et al*., 2011), whereas others showed no effect (Sartori *et al*., 2009; Davis *et al*., 2012).

Although the administration of eCG near the end of the FSH treatment protocol did not always improve the superovulatory response, it was not detrimental and raised some interest in its use to simplify the superstimulation protocol and to decrease the cost of the treatment, since eCG is usually less expensive than the pituitary extracts containing FSH. Therefore, we designed a study to evaluate the superovulatory response and embryo production in beef donors using twice daily FSH injections over 4 days or an alternative protocol in which the last 4 FSH treatments were replaced by a single injection of eCG (Barajas *et al*., 2019). Twelve (Experiment 1) and 18 (Experiment 2) mature Bonsmara donor cows were superstimulated twice at a 46-day interval in a crossover design. Follicular wave emergence was synchronized by the administration of estradiol-17β at the time of insertion of a P4-device and superstimulation was initiated 4 days later. Donors in the control group received 8 injections of FSH i.m. (total dose: 300 mg) in a twice-daily decreasing dosage schedule over 4 days, whereas donors in the FSH+eCG group received only the first 4 injections of FSH (total dose: 220 mg) and 48 h after initiating treatment, 800 IU of eCG i.m. in a single administration. All donors received PGF_2α_ i.m. with the eCG administration and again 12 h later. The P4-devices were removed in the AM of the next day. All cows received GnRH 24 hours after the removal of the P4-device and were inseminated with frozen/thawed semen from two bulls 12 and 24 hours later. Ova/embryos were collected and evaluated according to the IETS standards 7 days after the administration of GnRH. In Experiment 2, donors were treated only with FSH+eCG. The total dosage of FSH was 200 mg and the dosage of eCG was either 800 or 600 IU. Results of both experiments are presented in Table 1. In Experiment 1, the FSH (control) group produced a higher (P < 0.01) number of fertilized ova, but there were no differences in the number of transferable embryos. In Experiment 2, no differences were found between the FSH+800 eCG or FSH+600 eCG groups in any of the parameters evaluated. In conclusion, the replacement of the last 4 injections of FSH by a single administration of either 600 IU or 800 IU of eCG decreased the number of FSH treatments required in a superstimulation protocol without adversely affecting the production of transferable embryos.

**Table 1:** Embryo production (means ± SEM) in Bonsmara donors treated with FSH or FSH+eCG^£^.

	n	Total ova/embryos	Fertilized ova	Transferable embryos
Experiment 1				
FSH	12	11.7 ± 2.5	10.5 ± 2.3ª	5.7 ± 1.4
FSH+800 IU eCG	12	9.6 ± 1.5	6.8 ± 1.0^b^	5.3 ± 1.0
Experiment 2				
FSH+800 IU eCG	18	6.7 ± 0.7	5.4 ± 0.8	3.6 ± 0.7
FSH+600 IU eCG	18	6.1 ± 1.1	4.3 ± 1.0	3.7 ± 0.8

Different letters (a,b) within a column indicate significant difference (P < 0.05). ^£^Donors were treated with 8 intramuscular injections of FSH administered at 12 h intervals (FSH group) or the last 4 FSH treatments were replaced by a single intramuscular injection of eCG (FSH + eCG group).

## Manipulation of follicular development for *in vitro* embryo production (IVP)

The IVP of embryos, together with the technique of OPU, are reproductive biotechnologies that have advanced greatly in the last 10 years. This technology is highly developed in Brazil, where 57% of IVP embryos that are transferred in the world are produced (Viana *et al*., 2018). As indicated earlier, *Bos indicus* cattle have a higher number of follicles recruited per wave as compared to *Bos taurus* breeds and this has resulted in the recovery of a higher number of oocytes with OPU (Pontes *et al*., 2009; Baruselli *et al*., 2012; Watanabe *et al*., 2017). Likewise, field data from our laboratory has shown that Brahman-influenced synthetic breeds produce significantly more viable oocytes and transferable blastocysts following OPU/IVF than *Bos taurus* breeds (Bernal *et al*., 2016).

Several studies were designed to evaluate the effects of synchronizing follicular wave emergence and superstimulation on the number and quality of the COCs recovered by OPU and submitted to IVP (Ongaratto *et al*., 2015; Baruselli *et al*., 2016). The most important conclusions of these studies were: 1) Synchronizing follicle wave emergence prior to OPU increased the number of COCs obtained and blastocysts produced in *Bos taurus,* but not *Bos indicus* breeds; 2) Treatment with estradiol and P4 or the removal of the dominant follicle (DFR) were equally efficacious in the synchronization of follicular wave emergence for OPU; 3) Superstimulatory treatment with FSH increased the number and quality of COCs obtained by OPU in *Bos taurus* breeds, but not in *Bos indicus* breeds. In an experiment conducted in Brazil with Holstein donors (Vieira *et al*., 2014), all cows received a P4-device and 2 mg of EB (Day 0). Cows in the control group received no additional treatments, while cows in the FSH-treated group received twice daily treatments on Days 4 and 5 (total dose of 200 mg). On Day 7, the P4-device device was removed and the OPU was performed (40 h after the last FSH treatment). There were no differences between groups (P = 0.92) in the number of follicles that were aspirated per OPU session (17.2 ± 1.3 *vs* 17.1 ± 1.1 in the control and FSH-treated cows, respectively); however, COCs from FSH-treated cows yielded a higher blastocyst rate (34.5%, 89/258 *vs* 19.8%, 55/278, P < 0.001) and more transferable embryos per OPU session than the control group (3.0 ± 0.5 *vs* 1.8 ± 0.4, P = 0.02). It was concluded that superstimulation of Holstein donors with FSH before OPU increased the efficiency of IVP by increasing COC and embryo quality. In addition, non-lactating donors had a higher percentage of *in vitro* blastocyst development and produced more embryos per OPU session than lactating cows. In a later study, similar results were obtained when the four doses of FSH were replaced by a single i.m. injection of 200 mg of FSH diluted in a 0.5% hyaluronan solution (MAP-5, Vetoquinol; Vieira *et al*., 2015).

Two other studies were performed in Angus donors. In the first study (Ongaratto *et al*., 2011), administration of FSH resulted in a higher number of COCs obtained by OPU. In the second study (Ongaratto *et al*., *2019*), multiparous, non-lactating Angus cows, were randomly allocated into two treatment groups and treated twice in a cross-over design. Follicular wave emergence was synchronized with estradiol 17-β and progesterone, plus a P4-device. Four days later (Day 4) donors received either 160 mg FSH diluted in 4 ml of MAP-5 by a single i.m. injection or no FSH (Control group). COCs were obtained by OPU 72 h later (Day 7). Results are summarized in Table 2. The number of viable COCs was significantly higher in the FSH-treated donors than in controls.

**Table 2:** Mean (±SEM) numbers of total and viable cumulus oocyte complexes (COCs) recovered and number of blastocysts produced following superstimulation in Angus donors^£^.

Group	COCs		Blastocysts
	Total	Viable	Total
Single FSH (n = 9)	21.4 ± 2.4^a^	14.1 ± 1.6^a^	4.2 ± 0 .8
Control (n = 9)	15.9 ± 2.7^b^	10.6 ± 2.0^b^	2.7 ± 0.7
P Value	0.02	0.02	0.13

Different letters (a,b) within a column indicate significant difference (P < 0.05). ^£^Donors were treated with 5 mg estradiol 17-β and 50 mg of progesterone i.m. plus a P4-device on Day 0, followed by either 160 mg Folltropin-V diluted in 4 ml of MAP-5 by a single i.m. injection (Single FSH group) or no FSH (Control group) on Day 4. OPU was performed in both groups on Day 7.

Although administration of FSH prior to OPU has been a common practice for increasing the numbers of follicles available for OPU, most studies have adopted the conventional twice daily treatments with FSH, with either positive results in the number of COCs collected and embryos produced (Ongaratto *et al*., 2011; Blondin *et al*., 2012; Vieira *et al*., 2014; 2015) or no effect on COC or embryo production in Holstein cows (Oliveira *et al*., 2016). Obviously, the possibility of giving a single FSH injection instead of four prior to OPU in genetically superior animals is critically important for the widespread application of this technology in commercial herds, where personnel are not as familiar with intensive treatment protocols as producers working with purebred cattle.

## Strategies for the application of *in vivo* and *in vitro* embryo transfer in commercial operations

The commercial embryo transfer industry began in North America in the early 1970s, and the technology soon spread to South America (Bó and Mapletoft, 2014). Brazil and Argentina have consistently ranked in the top five countries outside North America and Europe in the production IVD embryos. Viana (2018) has reported recently that more than 992,289 IVP and 495,054 IVD bovine embryos were produced worldwide in 2017. North America accounted for more than 59% (292,755) of the IVD embryos, while South America only accounted for 10% (49,230). On the other hand, the distribution of IVP embryos were similar in North (475,696; 48%) and South (453,685; 46%) America. This is the first report in which North America produced more IVP than IVD embryos, whereas in South America the number of IVP embryos has been higher than the number of IVD embryos for more than 10 years.

The application of a successful program using IVD or IVP embryos not only relies on a robust IVP system, but also on the implementation of a successful embryo transfer program. Nutrition, management and efficiency in the synchronization of estrus and ovulation are among the factors that affect the use of these technologies (Mapletoft and Bó, 2016). To avoid limitations associated with estrus detection, treatments that synchronize the time of ovulation in recipients, which were developed originally for fixed-time AI (FTAI), have been utilized for fixed-time embryo transfer (FTET; Bó *et al*., 2002a, 2012a). These treatments are generally divided into those that are GnRH-based (Ambrose *et al*., 1999) and those that are estradiol-based (Bó *et al*., 2002) and are selected depending on the availability of the hormones in different countries. In either case, the recipient protocols include the insertion of a P4-device for 7 or 8 days (Hinshaw, 1999; Bó *et al*., 2002b).

Estradiol and P4- (estradiol/P4) based treatments are the most commonly used protocols to synchronize follicle wave emergence and ovulation of recipients in South America (Baruselli *et al*., 2010). The simplified protocol used most commonly consists of insertion of a P4-device and the administration of 2 mg EB on Day 0, and PGF_2α_ at the time of insertion and removal of the P4-device if it is impregnated with >1 g of P4 and only at P4-device removal when it contains <1 g of P4. The P4-device is usually removed on Day 7 or 8 and 300 or 400 IU of eCG are administered at that time (Bó *et al*., 2002a). Ovulation is induced by the administration of 0.5 or 1 mg of estradiol cypionate (ECP) at the time of P4-device removal and all recipients with a corpus luteum (CL) 9 days later receive an embryo (i.e., 7 days after the expected time of estrus; Baruselli *et al..* 2010, 2011; Bó *et al*., 2012b). Overall, 75 to 85% of the recipients treated with this protocol receive an embryo; P4 concentrations are high at the time of embryo transfer and pregnancy per embryo transfer (P/ET) range from 40 to 60%, when both embryos and recipients are of high quality (reviewed in Bó *et al*., 2002a; Baruselli *et al*., 2010, 2011).

Recent studies have suggested that increasing the interval from P4-device removal to FTAI may improve pregnancy per AI (P/AI) in a GnRH-based protocol (named 5-day CoSynch+CIDR) (Bridges *et al*., 2008) or estradiol/P4-based treatments (named J-Synch protocol; Bó *et al*., 2016). In both protocols, a second GnRH is administered 72 hours after the removal of the P4-device (prolonged proestrus). The benefits associated with the prolonged proestrus were a prolonged exposure to estradiol prior to ovulation and an increased ability of the uterus to support embryo development (reviewed in Bó and Cedeño, 2018).

**Figure 1:**
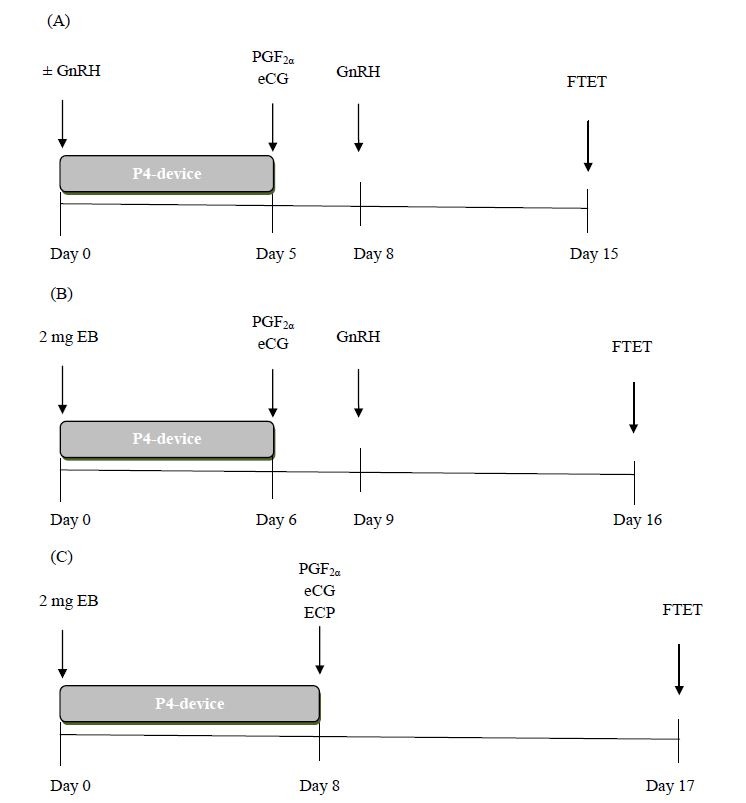
Recommended protocols for FTET in bovine recipients. A) Modified 5-day GnRH+P4 device protocol (P4 device on Day 0, PGF2α and eCG on Day 5 and GnRH on Day 8). B) J- Synch protocol (P4 device and EB on Day 0, PGF2α and eCG on Day 6 and GnRH on Day 9). C) Conventional ECP protocol (P4 device and EB on Day 0, PGF2α, eCG and ECP on Day 8) If estrus detection is implemented with tail patches or tail paint, recipients are observed 72 h after P4 device removal in protocols A and B and 54 h after P4 removal on protocol B.

Using a modified 5-day CoSynch+CIDR protocol (no GnRH at P4-device insertion, a single injection of PGF2α at P4 removal on Day 5 and GnRH on Day 8), Sala *et al.* (2016) reported similar P/ET rates with IVP embryos as in recipients synchronized with two PGF_2α_ treatments 14 days apart and estrus detection. Furthermore, Menchaca *et al.* (2015, 2016) reported higher pregnancy rates in beef recipients receiving Holstein IVP embryos and synchronized with the J-Synch protocol as compared to the conventional estradiol/P4 protocol in which ECP was given at P4-device removal. Although embryos can be transferred at a fixed time, without estrus observation, optimal P/ET and calving rates were obtained when tail paint was used to detect estrus and only recipients with their tail paint rubbed-off (i.e., in estrus) received embryos 7 days later (reviewed in Bó and Cedeño, 2018). The recommended protocols for FTET in recipients are shown in Figure 1.

## Embryo Transfer in commercial beef herds

Although the numbers of embryos produced have increased over the years in several regions of the world (Viana, 2018), the main bottleneck for the widespread application of this technology has been the availability of recipients and the intensive management needed for embryo transfer in commercial herds. This problem has been changing as a consequence of the widespread application of successful FTAI in commercial herds. Therefore, in farms where personnel are already familiar with the application of synchronization and re-synchronization of ovulation for FTAI, the application of an embryo transfer program with the normal management of the cow herd seems feasible. Lactating cows and heifers can be synchronized at the beginning of the breeding season to receive IVD or IVP embryos and then resynchronized and inseminated or just simply exposed to clean-up bulls during the remainder of the breeding season. A study was designed to examine pregnancy rates in *Bos indicus* cows that were synchronized for FTAI or FTET at the beginning of the breeding season (Martins *et al*., 2014). In this experiment, 634 lactating Nelore cows were randomly assigned to one of four treatment groups: 2 FTAI (n = 160), 2 FTET (n = 152), 1 FTAI followed by 1 FTET (FTAI/FTET; n = 160) and 1 FTET followed by 1 FTAI (FTET/FTAI; n = 158). All animals were treated with a P4-device for 8 days, EB on Day 0, and PGF_2α_, eCG and ECP on Day 8. Cows undergoing FTAI were inseminated 48 h after P4-device removal, whereas those receiving embryos were evaluated for the presence of CL 9 days after P4-device removal and those with a CL received IVP embryos. Cows in both groups were resynchronized using the same protocol that was used for the first service, 30 days after the first FTAI or 23 days after FTET. The post-partum period at the times of first service was 41.4 days for FTAI and 47.4 days for FTET and 82.8 days for FTAI and 89.8 days for FTET for the second service. Pregnancy diagnosis was performed by ultrasonography at 30 and 60 days of gestation. Pregnancy rates after the first service were higher (P < 0.01) in cows that were FTAI (59.4% and 59.4% for 2 FTAI and FTAI/FTET group respectively) than those that were FTET (31.7% and 32.7% for 2 FTET and FTET/FTAI groups, respectively). Similarly, pregnancy rates after the second service also differed (P = 0.06) among groups: 2 FTAI (50.8%), FTAI/FTET (40.6%) FTET/FTAI (51.9%) and 2 FTET (35.0%). Finally, the cumulative pregnancy rate (first + second service) was higher in the groups that receive 2 FTAI (80.0%) than those receiving 2 FTET (55.8%); pregnancy rates in the other groups that received the combined techniques were intermediate and did not differ (FTAI/FTET: 75.6% and FTET/FTAI: 66.5%, respectively). The conclusion of this study was that although the use of two consecutive FTET had a lower cumulative pregnancy rate than two consecutive FTAI, the association between FTAI and FTET programs can be considered as an alternate strategy to increase number of offspring from embryo transfer.

Other approaches have been implemented in the field. In one study (Bó, personal communication), an embryo transfer program was implemented in an extensive Hereford herd in Southern Argentina. In this herd, cows were synchronized with a conventional estradiol/P4-based synchronization protocol for FTET (n = 62) or were FTAI at the beginning of the breeding season (n = 300). In the FTET 49 cows (79.0%) had CL 9 days after P4-device removal and received IVD frozen/thawed embryos. The day after FTET all cows were exposed to clean up bulls for the remaining of the breeding season. Twenty cows (40.8%), were pregnant following FTET and the pregnancy rate to FTAI was 54% (162/300; P < 0.05). However, the overall pregnancy rate at the end of the breeding season was 91.0% and did not differ among groups.

**Figure 2:**
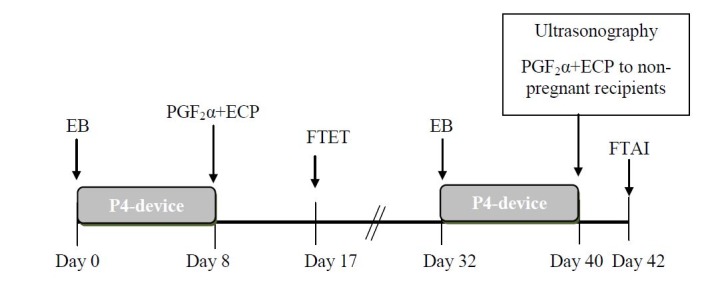
Simple proposal for FTET and resynchronization with FTAI in beef cattle. All cows treated on Day 0 (transferred or not on Day 17) are resynchronized on Day 32. All those recipients not pregnant by Day 30 of gestation (Day 40 of this protocol) are FTAI two days later.

These previous studies are examples of the many approaches that can be implemented using FTAI and FTET in commercial beef herds. Most of the protocols were designed for synchronization and resynchronization and have been published elsewhere (for recent reviews see Bó *et al*., 2016 and Baruselli *et al*., 2017). Two of the protocols for re-synchronization are called Resynch 22 and Resynch 14 (Baruselli *et al*., 2017). In the Resynch 22, cows receive 2 mg EB and heifers 1 mg EB at P4-device insertion on Day 22 after FTAI. Pregnancy diagnosis is performed at device removal (Day 30) and non-pregnant animals receive PGF_2α_ and ECP and are inseminated on Day 32. The Resynch 14 protocol involves the use of color Doppler ultrasonography for the detection of pregnancy based on the vascularization and size of the CL on Day 22 after the first AI. The resynchronization treatment starts 14 days after FTAI with the re-insertion of a used P4-device and the administration of 100 mg P4 IM (Rezende *et al*., 2016) at the same time. Cows are scanned with Doppler ultrasonography for pregnancy at P4-device removal (Day 22) and non-pregnant animals receive PGF_2α_ and ECP and are inseminated on Day 24. A proposed program using FTET and Resynch 22 for FTAI is illustrated in Figure 2.

## Use of embryo transfer in dairy herds

Traditionally, embryo transfer has been implemented in dairy herds to reproduce animals of high genetic merit, but it has also been shown to improve the reproductive performance of high producing commercial herds. The main reason for the potential improvement is the higher fertility reported after embryo transfer in cows experiencing heat stress (Putney *et al*., 1989; Ambrose *et al*., 1999; Rodrigues *et al*., 2004, 2007a, 2007b; 2011; Baruselli *et al*., 2010) and those diagnosed as repeat-breeders (Rodrigues *et al*., 2007b; 2010; Dochi *et al*., 2008; Ferreira *et al*., 2011; Stewart *et al*., 2011). In a retrospective study that was performed using lactating Holstein cows, conception rates were higher across the year in cows receiving embryos as compared to those that were AI, but the differences were more pronounced in the warmer months of the year (November through April in the southern hemisphere) (Rodrigues *et al*., 2007a). In a subsequent study, embryonic loss between 30 and 60 days of pregnancy was also compared retrospectively in lactating Holstein cows subjected to AI or embryo transfer during summer and winter months (reviewed by Baruselli *et al*., 2011). Although pregnancy loss was higher for embryo transfer than for AI, cows receiving embryos had higher pregnancy rates after 60 days than those that were AI. Therefore, a useful management tool to maintain high pregnancy rates throughout the year would be to produce embryos during the cooler months and use them in embryo transfer during the periods of heat stress.

Another alternative that has tremendous application is to use embryos in repeat breeder cows. Repeat breeder cows are usually defined as cows that do not become pregnant over a period of time (usually after 3 or 4 unsuccessful breedings) that do not have any apparent abnormality that can be diagnosed by a veterinary examination. In a recent study, the transfer of embryos to repeat breeders resulted in increased pregnancy rates compared to AI, without differences in embryo/fetal losses between 30 and 60 days (Ferreira *et al*., 2010). In another retrospective study (Rodrigues *et al*., 2007b), conception rates in repeat breeder Holstein cows were greater after transfer of IVD embryos (41.7%; 1609/3858) than after AI (17.9%; 1019/5693), supporting the notion that the fertility problem in some repeat-breeders may be associated with oocyte quality and/or failure of early embryo development. Other reports have also shown significant improvements in pregnancy rates using IVD (Son *et al*., 2007; Dochi *et al*., 2008) or IVP embryos (Block *et al*., 2010) in repeat breeder cows. The strategy that will have the greatest impact on the fertility of the herd is to use FTET (without estrus detection; Rodrigues *et al*., 2010) and IVP or IVD embryos produced with sexed semen, to increase the number of female calves born in the herd. Certainly, IVP is an efficient method for using sexed semen (Wheeler *et al*., 2006; Pontes *et al*., 2010; Rasmussen *et al*., 2013; Pellegrino *et al*., 2016) and multiple embryos can be produced using a single straw of semen.

## Concluding remarks

The use of protocols that control follicular development and ovulation have the advantage of allowing the widespread application of assisted reproductive technologies. The treatments used to synchronize follicle wave emergence for superovulation by many practitioners around the world have proven to be practical and easy to perform by field staff. Lengthened the superstimulation protocol is also an interesting alternative to produce embryos *in vivo*, especially in cows with reduced antral follicle populations, because the time necessary for all growing follicles to acquire the ability to ovulate is extended. With respect to the IVP of embryos, cattle with *Bos indicus* influence adapt very well to this technology because they have a high antral follicle population and, consequently, more COCs are obtained by OPU than in *Bos taurus* breeds. In *Bos taurus* donors, synchronization of follicular wave emergence and the use of FSH have resulted in a greater number of COCs per OPU and the IVP of a higher percentage of blastocysts. Nowadays *in vitro* and *in vivo* production of embryos can be combined with efficient synchronization and FTET programs that are easily implemented in the field and permit the inclusion of commercial beef herds in embryo transfer programs, combined with FTAI or simply with clean-up bulls. Although the embryo transfer technology has been used in dairy herds for many years, primarily for genetic improvement in a limited number of cows the technology can now be used to resolve reproductive problems such as the reduced fertility observed during the summer months and in repeat breeder cows.
